# A Three-Hybrid System to Probe *In Vivo* Protein-Protein Interactions: Application to the Essential Proteins of the RD1 Complex of *M. tuberculosis*

**DOI:** 10.1371/journal.pone.0027503

**Published:** 2011-11-08

**Authors:** Megha Tharad, Sachin Kumar Samuchiwal, Kuhulika Bhalla, Anamika Ghosh, Krishan Kumar, Sushil Kumar, Anand Ranganathan

**Affiliations:** Recombinant Gene Products Group, International Centre for Genetic Engineering and Biotechnology, Aruna Asaf Ali Marg, New Delhi, India; Loyola University Medical Center, United States of America

## Abstract

**Background:**

Protein-protein interactions play a crucial role in enabling a pathogen to survive within a host. In many cases the interactions involve a complex of proteins rather than just two given proteins. This is especially true for pathogens like *M. tuberculosis* that are able to successfully survive the inhospitable environment of the macrophage. Studying such interactions in detail may help in developing small molecules that either disrupt or augment the interactions. Here, we describe the development of an *E. coli* based bacterial three-hybrid system that can be used effectively to study ternary protein complexes.

**Methodology/Principal Findings:**

The protein-protein interactions involved in *M. tuberculosis* pathogenesis have been used as a model for the validation of the three-hybrid system. Using the *M. tuberculosis* RD1 encoded proteins CFP10, ESAT6 and Rv3871 for our proof-of-concept studies, we show that the interaction between the proteins CFP10 and Rv3871 is strengthened and stabilized in the presence of ESAT6, the known heterodimeric partner of CFP10. Isolating peptide candidates that can disrupt crucial protein-protein interactions is another application that the system offers. We demonstrate this by using CFP10 protein as a disruptor of a previously established interaction between ESAT6 and a small peptide HCL1; at the same time we also show that CFP10 is not able to disrupt the strong interaction between ESAT6 and another peptide SL3.

**Conclusions/Significance:**

The validation of the three-hybrid system paves the way for finding new peptides that are stronger binders of ESAT6 compared even to its natural partner CFP10. Additionally, we believe that the system offers an opportunity to study tri-protein complexes and also perform a screening of protein/peptide binders to known interacting proteins so as to elucidate novel tri-protein complexes.

## Introduction

*M. tuberculosis* continues to spread and kill millions despite the availability of vaccines and drugs that can combat the pathogen [1]. Recent emergence of strains that are resistant to all of the current front-line Tuberculosis (TB) drugs has caused world-wide alarm, and there is an urgent need for the development of new and more efficient drugs and simple diagnostic tools to help fight the scourge [2], [3].

A characteristic feature of *M. tuberculosis* is its ability to remain dormant in the host for years [4]. It enters the human body through the respiratory tract, gets engulfed by the phagocytic cells and is carried across the alveolar epithelium to the lungs, where a dynamic process of sequestration and infection of fresh phagocytic cells by the pathogen and formation of granuloma takes place [5], [6]. During this entire process, and also during the stage of progressive infection, a series of protein-protein interactions within the pathogen as well as at the host-pathogen interface takes place [7]–[9]. This enables the pathogen to adapt to the inhospitable changes in the immediate environment and eventually get established as a persistent infection.

Several studies have been carried out to understand the complex nature of these protein-protein interactions. Two mycobacterial secretory proteins, SapM [10] and PtpA [11] have been shown to directly interfere with the host physiological processes, resulting in maturation arrest of the mycobacterial phagosome. The RD1 region encoded virulence determining factors, CFP10 and ESAT6 are another set of secretory proteins that are of importance in this context. ESAT6 and CFP10 form a strong 1∶1 heterodimeric complex which is recognized and subsequently secreted with the aid of a specialized secretory system called the EsxI system [12]–[15]. Recent studies have shown that the EsxI secretory system is complex in nature and involves several proteins that assist in the secretion of its substrates CFP10 and ESAT6. An EsxI secretory system protein Rv3871 is a cytosolic ATPase that has been shown to bind to the C-terminal of CFP10 in the ESAT6 : CFP10 complex and escort it to Rv3870, the membrane-bound component of the EsxI system protein, and thereby allow its secretion [14]. The system has several other substrates that are co-secreted and are mutually dependent upon each other for secretion, such that the inhibition of secretion of any of these substrates can affect the secretion of the rest of the substrates [16]. Through functional and comparative genomic studies, it is now known that the secretion of ESAT6 and CFP10 is crucial for stimulating host immunogenicity [17] while imparting a fully virulent phenotype to *M. tuberculosis* [13], [18], [19]. ESAT6 has been known to be involved in the dissemination of the pathogenic mycobacterium within the host body and shown to directly interact with the host epithelial cell protein MMP9 to induce granuloma formation [20], [21].

Given the fact that protein-protein interactions are fundamental for pathogenesis, disruption of any of these interactions can have a debilitating effect on the normal sustenance of the pathogen within the host system [22], [23]. Disrupting protein-protein interactions using a bacterial three-hybrid system, therefore, provides a viable approach towards deciphering the interaction mechanisms involved, besides presenting new avenues for pharmacological applications. Conventional strategies for disruption of known and crucial protein-protein interactions involve the use of small molecules that can enter the infected cell in question and perform the disruption [22], [24]. These molecules are, more often than not, representatives from a chemically synthesized combinatorial library or from defined/undefined compound libraries collected over decades. In contrast, using *de novo* proteins and peptides as ‘interactors’ offers a feasible alternative to this approach as it allows us to sift through the protein space and explore a myriad of proteins from a given *de novo* protein library, so as to isolate peptides/proteins that may interact and then possibly disrupt a given protein-protein interaction [25], [26]. To make use of such an approach, what is required is a viable system that can express the genes corresponding to the *de novo* peptides or proteins, and then present such peptides and proteins to an interacting protein complex at the first place.

Ideally, such a system should (a) enable the expression of three different genes in a single bacterial system in a regulated manner, (b) facilitate simple detection of the effect of association of a third protein on two interacting proteins, and (c) efficiently allow the use of *de novo* peptide libraries in the three-hybrid set-up. In this report, we describe the development and functional analysis of an *E. coli* based bacterial three-hybrid system that addresses all of the above-mentioned concerns.

## Results

### Construction of a bacterial three-hybrid system

**Figure 1:**
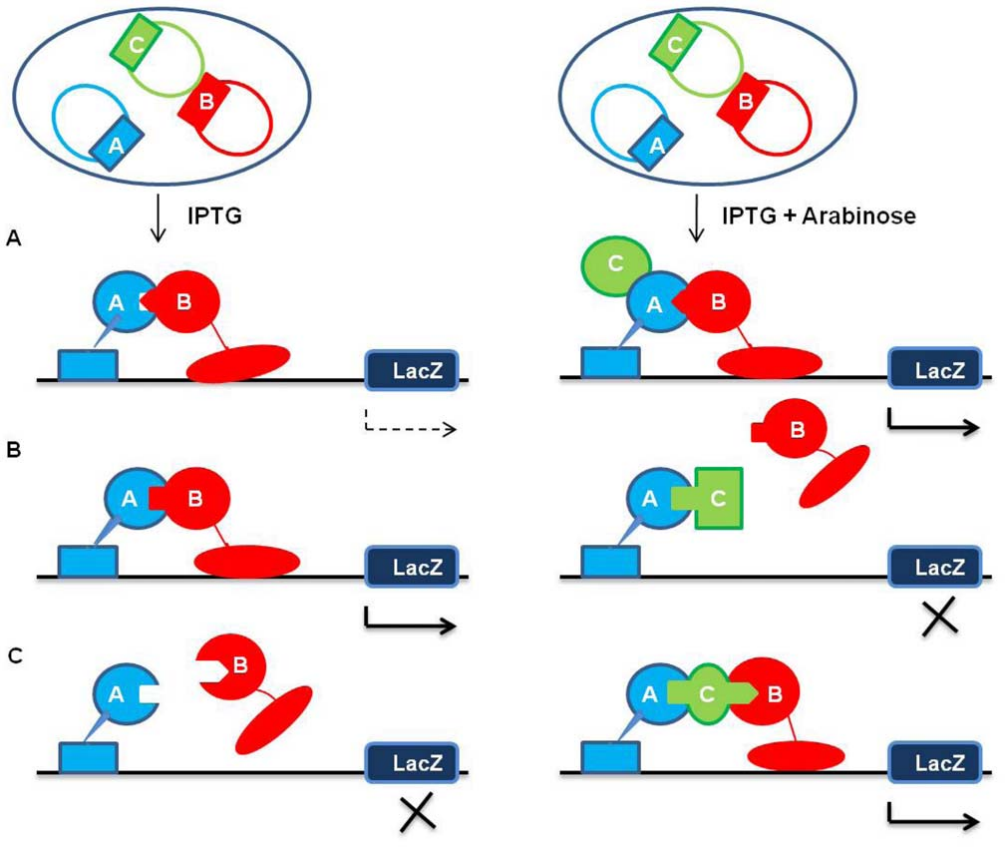
Schematic representation of the bacterial three-hybrid system. The diagram represents the effect of expression of a third protein under arabinose induction on the interaction of IPTG-inducible bait and target proteins. Here A and B represent the bait and target proteins respectively, whereas C represents the protein expressed by the gene cloned in pMTSA vector. (A) Protein C acting as an inducer of interaction between proteins A and B. (B) Protein C acting as a disruptor of interaction between proteins A and B. (C) Protein C acting as a mediator of interaction between proteins A and B.

The three-hybrid system was decided to be based on an original transcriptional activation dependent bacterial two-hybrid system that was introduced by Dove and coworkers [27], [28]. This two-hybrid system has been used extensively over the past decade to study and validate interactions between any two given proteins. The ‘read-out’ of a successful interaction is visual (appearance of blue-colored colonies) as it is based on the lacZ-Xgal ‘blue-white’ selection, and the strength of the interaction can be easily monitored using the ‘lacZ activity’ assay [28]. We decided to build on this system and develop a simple method wherein a ‘blue reporter’ strain (indicative of a successful protein-protein interaction), carrying the interacting bait and target proteins could be transformed with a vector carrying a gene that would express a third protein. Upon induction, the expression of this third protein could affect the bait and target protein interaction and bring about its manifestation in the form of an altered level of reporter gene transcription. In other words, if the third protein were to augment the target and bait interaction, the blue color would become more intense; on the other hand, if the said interaction was disrupted by the third protein, the effect would be a decrease in lacZ enzyme activity, leading to a change in color from blue to white. The three-hybrid system and its potential uses are shown schematically in Figure 1A, 1B and 1C.

For the construction of the bacterial three-hybrid system, a new vector plasmid was required, that could coexist in the reporter strain along with the bait and the target plasmids, and carry a promoter that would allow differential regulation of expression of the third gene. A novel streptomycin-resistant pMTSA vector was thereby constructed that had all the elements required for its viability in the newly developed system (see Materials and Methods). The integrity of the vector was confirmed by restriction enzyme digestion and sequencing. To check for its functionality, *Rv3871* gene was cloned in the pMTSA vector and the resulting plasmid used to transform the bacterial two-hybrid ‘R1’ strain. A robust expression of the Rv3871 protein under arabinose induction was observed (Figure S1). The system was now ready to be used for the study of ternary protein complexes.

### The CFP10, Rv3871, ESAT6 tri-protein complex

**Figure 2:**
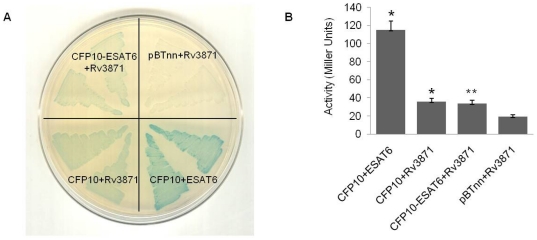
CFP10 : Rv3871 protein-protein interaction. (A) Bacterial two-hybrid X-Gal plate showing co-transformants: CFP10pBTnn + ESAT6pTRGnn (positive control); CFP10pBTnn + Rv3871pTRGnn; CFP10-ESAT6pBTnn + Rv3871pTRGnn; and pBTnn + Rv3871pTRGnn (negative control). “+" denotes the fact that two or more proteins (or plasmids) are present together, either within a strain or *in vitro*. Two different colonies of each co-transformant were patched. (B) Quantitative analysis by liquid β-galactosidase assay. All interactions were found to be statistically significant (*, P<0.001; **, P<0.05).

Interactions between the *M. tuberculosis* EsxI proteins CFP10 and ESAT6, as well as between CFP10 and Rv3871 have recently been elucidated in seminal studies by Cox and co-workers and a model for the export of ESAT6 protein has been proposed [14]. According to this model, the C-terminal end of CFP10 protein interacts with Rv3871, triggering the eventual export of the CFP10 : ESAT6 heterodimeric complex itself (the punctuation mark “:” denotes an interacting protein complex.) We were interested in studying this tri-protein complex in order to ascertain whether ESAT6 has a role in CFP10 : Rv3871 protein-protein interaction. *In vivo* validation of the interaction between CFP10 and ESAT6, as well as CFP10 and Rv3871 was first achieved using the bacterial two-hybrid system. Appearance of dark blue color in the ‘CFP10 : ESAT6 strain’ and lighter blue color in the ‘CFP10 : Rv3871 strain’ indicated the relative strength of interaction (Fig 2A). The results were in accordance with previously published reports [14] and also ensured that the folding and the subsequent interaction of the concerned proteins were appropriate in the bacterial strain. *In vitro* analysis of the CFP10 : ESAT6 and CFP10 : Rv3871 protein-protein interactions was carried out by Far-Western Dot Blot assay (Figure S2B) using purified CFP10-GST (Glutathione S-transferase), Rv3871-His and ESAT6-His proteins (Figure S2A; the punctuation “-” denotes a fusion protein or a protein fused to a peptide-tag). Quantification of the density of blot color was done by Spot Densitometric analysis (Figure S2C). Observation of a strong interaction between CFP10 : ESAT6 and a weak but significant interaction between CFP10 : Rv3871 helped us further confirm the concerned protein-protein interactions.

Before commencing our three-hybrid studies, we decided to first profile the interaction of Rv3871 with CFP10-ESAT6 and ESAT6-CFP10 fusion proteins using the two-hybrid system. We wanted to investigate whether, within the limitations of this ‘two protein’ system, we could establish if Rv3871 interacted more strongly with CFP10 when the latter was in fusion with its natural partner ESAT6 than when CFP10 was on its own. It is known that CFP10, by forming a 1∶1 heterodimeric complex with ESAT6, undergoes a conformational change and attains higher thermodynamic and biochemical stability [29]. It is highly probable, therefore, that the interaction of Rv3871 with CFP10 is affected by the presence or absence of ESAT6.

To avoid any discrepancy in terms of conformation, both fusion orientations i.e. CFP10-ESAT6 and ESAT6-CFP10 were constructed and analyzed. Interaction strength of Rv3871 with the fusion constructs was found to be comparable to that with CFP10 alone (Fig 2A; Figure S3A). Corroboration of all visual observations was carried out by the quantitative liquid β-galactosidase assay (Fig 2B; Figure S3B). Rv3871 was found to bind both fusion constructs with equal strength, notwithstanding the fact that in one of the fusion constructs – CFP10-ESAT6 – the C-terminal end of CFP10 is covalently tethered and not free to interact with Rv3871. This may point to the fact that factors other than the C-terminal chain of CFP10 are involved in the interaction, although this could also be due to the fact that the flexible N-terminal of ESAT6 acts as a linker allowing the C-terminal of CFP10 to be available for other binding partners. Clearly, using a two-hybrid approach was not going to be sufficient for studying tri-protein complexes even though two of the constituent proteins formed a natural association with each other.

**Figure 3:**
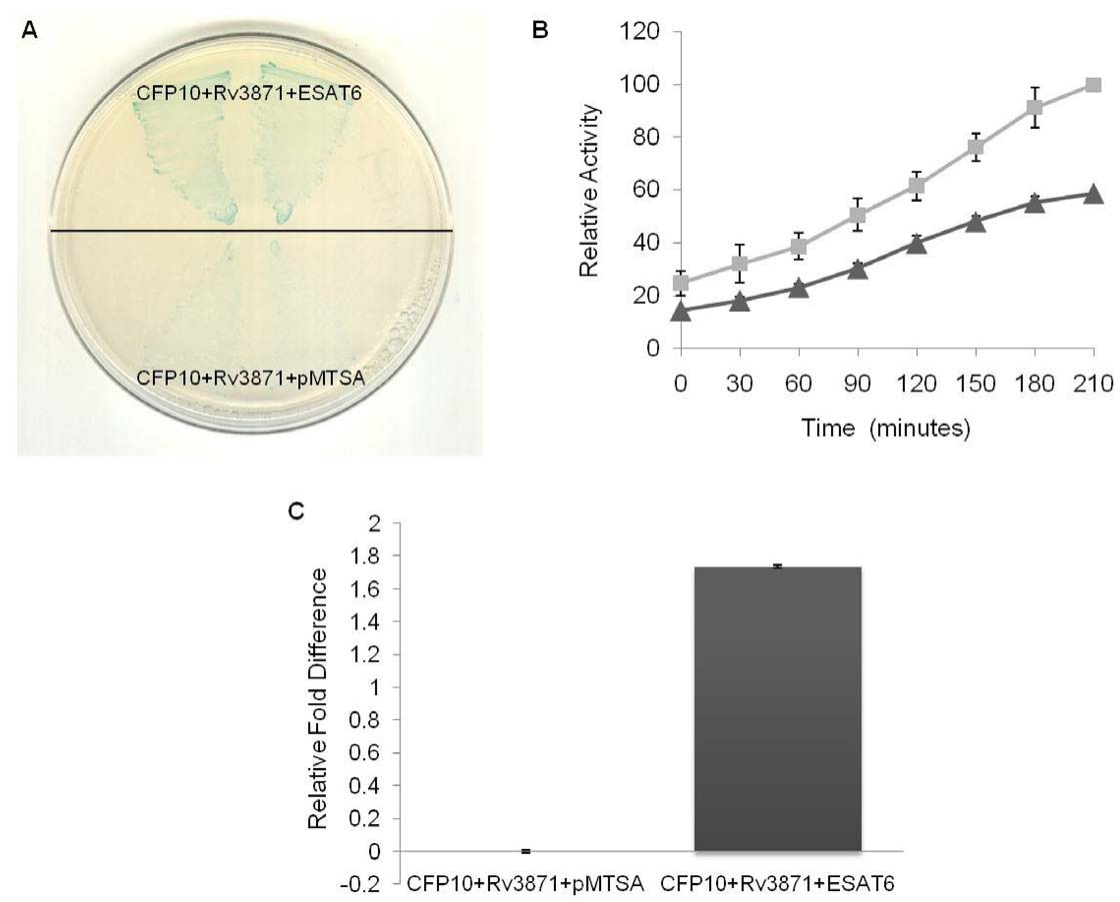
Representation of the CFP10 : Rv3871 : ESAT6 interaction in bacterial three-hybrid system. (A) X-Gal Arabinose indicator plate showing triple co-transformants: CFP10pBTnn + Rv3871pTRGnn + ESAT6pMTSA; and CFP10pBTnn + Rv3871pTRGnn + pMTSA. Two separate colonies from each co-transformant plate were picked and patched. (B) Time course liquid β-galactosidase assay: Relative β-galactosidase activity of the triple co-transformants (▴) CFP10pBTnn + Rv3871pTRGnn + pMTSA; and (▪) CFP10pBTnn + Rv3871pTRGnn + ESAT6pMTSA is plotted against time-points of bacterial culture growth, with 0 time-point being the point of arabinose induction. The graph is the average of three independent assays and standard deviation is represented as error bars. (P<0.05 at all time points beyond 60 minutes). (C) Relative fold change of *lacZ* gene transcripts in Bacterial three-hybrid system: Approximately two-fold increase in *lacZ* gene transcription is observed in comparison to negative control when CFP10 and Rv3871 interact in the presence of ESAT6 in the three-hybrid system. (*, P<0.01).

Utilizing the bacterial three-hybrid system, that enables three proteins to be expressed simultaneously yet exclusive to each other, we believed, was therefore ideally suited for such tri-protein complex studies. Firstly, it would allow their interactions to mimic the natural complex more closely. Additionally, in the context of CFP10, ESAT6 and Rv3871, the three proteins would be allowed to express independently in a system, rather than as fusion constructs. With this in mind, a three-hybrid approach was adapted. The ‘blue reporter strain’ carrying CFP10pBTnn and Rv3871pTRGnn plasmids was transformed with ESAT6pMTSA (test) or pMTSA (control) plasmids separately and plated on X-Gal arabinose indicator plates. Deep blue-colored colonies in the strain carrying ESAT6pMTSA, whereas lighter blue color in the strain carrying empty pMTSA plasmid, were observed (Fig 3A). Random colonies from each plate were picked and checked for the presence of all the three plasmids.

For a quantitative study of the above observation, time-dependent liquid β-galactosidase assay was carried out. A graph of β-galactosidase activity of each sample plotted against time showed a significant difference in the activity of test and control samples (P<0.05). The enzyme activity in the strain carrying ESAT6pMTSA was found considerably higher than that in the control strain (Fig 3B).

An RT-PCR based analysis was used to determine the level of transcription of the *lacZ* reporter gene in the test and control strains. The results reaffirmed the quantitative readout of the β-galactosidase assays. Approximately two-fold increase in *lacZ* gene transcription was obtained in the CFP10 : Rv3871 : ESAT6 strain compared with the negative control (Fig 3C). An RT-PCR analysis to check the level of expression of CFP10 and Rv3871 was also conducted. We found no difference in transcription levels in the test and control strains (Figure S4) demonstrating that the difference in the extent of interaction indicated by the strength of blue color and the *lacZ* gene expression was solely because of the presence or absence of ESAT6 in the three-hybrid strain and not because of the differential expression of CFP10 and Rv3871.

### The ESAT6, HCL1, CFP10 tri-protein complex

As previously mentioned, the interaction between ESAT6 and CFP10 has been well established and efforts to discover binders to these ‘virulence determinants’ of *M. tuberculosis* are underway [12], [29], [30]. Recently, Kumar *et al* have reported another peptide HCL1, isolated from human lung cDNA library, that shows strong interaction with ESAT6 of both *M. tuberculosis* as well as *M. smegmatis,* with its *in vivo* expression severely impairing *M. tuberculosis* growth [31]. We decided to test the efficacy of the three-hybrid system using proteins CFP10, ESAT6 and HCL1. Verification of these interactions was first achieved by the standard bacterial two-hybrid assay which showed strong interaction between ESAT6 and CFP10, as well as between ESAT6 and HCL1 (Figure S5A). A liquid β-galactosidase assay estimation showed that the interaction between ESAT6 and CFP10 was a little stronger compared to that between ESAT6 and HCL1 (Figure S5B). Further analysis using *in vitro* Far-Western Dot Blot assay showed that HCL1 interacted strongly with ESAT6 while not at all with CFP10 (Figure S5C).

To explore how these three proteins would behave when all three of them are present in the same system, ESAT6 and CFP10 were taken as bait and target proteins respectively and the resulting ‘blue strain’ was transformed with HCL1pMTSA (test) or pMTSA (control) plasmids. No considerable variation in blue color of the colonies in test and control plates was observed. The observation was confirmed by liquid β-galactosidase assay (data not shown). This meant that, upon arabinose induction and expression, HCL1 was not able to disrupt ESAT6 : CFP10 interaction.

**Figure 4:**
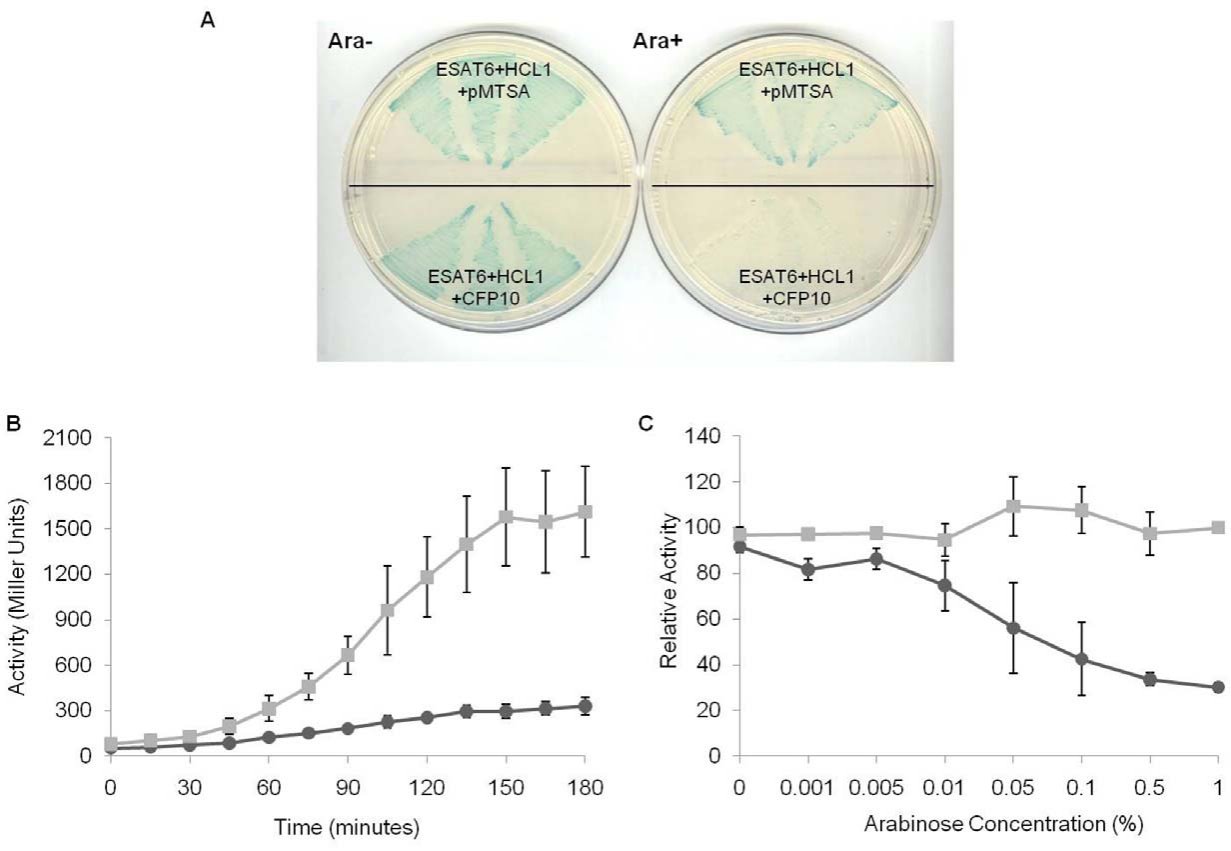
Representation of ESAT6 : HCL1 protein-protein disruption by CFP10 in bacterial three-hybrid system. (A) X-Gal indicator plate with and without arabinose patched with ESAT6pBTnn + HCL1pTRGnn + pMTSA; and ESAT6pBTnn + HCL1pTRGnn + CFP10pMTSA. Blue colony turns white when CFP10 is allowed to express in the presence of 1% arabinose. (B) Time course liquid β-galactosidase assay: β-galactosidase activity of above triple co-transformants: (▪) ESAT6pBTnn + HCL1pTRGnn + pMTSA; and (•) ESAT6pBTnn + HCL1pTRGnn + CFP10pMTSA is plotted against time-points of bacterial culture growth with 0 time-point being the point of arabinose induction. Standard deviation of the activities obtained in three separate assays is shown by error bars. (P<0.05 at all time-points beyond 60 minutes) (C) Arabinose gradient liquid β-galactosidase assay: Relative β-galactosidase activity of triple co-transformants (•) ESAT6pBTnn + HCL1pTRGnn + CFP10pMTSA; and (▪) ESAT6pBTnn + HCL1pTRGnn + pMTSA is plotted against arabinose concentration. The graph is the average of three separate assays and standard deviation is represented as error bars. (P<0.05 at all arabinose concentrations beyond 0.01%).

To investigate the possibility whether CFP10, instead, could disrupt the interaction between ESAT6 and HCL1, the ‘blue strain’ carrying ESAT6pBTnn and HCL1pTRGnn plasmids was transformed with CFP10pMTSA (test) or pMTSA (control) plasmids. Herein, an interesting observation on the X-Gal arabinose indicator plate was made. The colonies on control plate remained blue, while those on test plate had turned white (Fig 4A). Liquid β-galactosidase assay showed a five-fold decrease in β-galactosidase activity in test sample as compared to the control sample (Fig 4B). No indication of the CFP10 expression having a toxic effect on cells was observed. Transforming the ESAT6 : HCL1 blue strain with a dummy (unrelated protein) non-interactor HLL7pMTSA plasmid gave same results as the negative control (Figure S6A, B).

Our next objective was to check the effect varying concentrations of the inducer arabinose may have over the ESAT6 : HCL1 protein-protein disruption by CFP10. An arabinose gradient liquid β-galactosidase assay was performed. It was hypothesized that with increasing concentration of arabinose, expression of CFP10 in the strain would increase, which, in turn, should enhance the extent of disruption of ESAT6 : HCL1 interaction. A graph of enzyme activity versus arabinose concentration clearly showed more than three-fold decrease in activity of the test sample with increasing arabinose concentration, while no considerable change in activity in case of negative control was observed (Fig 4C).

**Figure 5:**
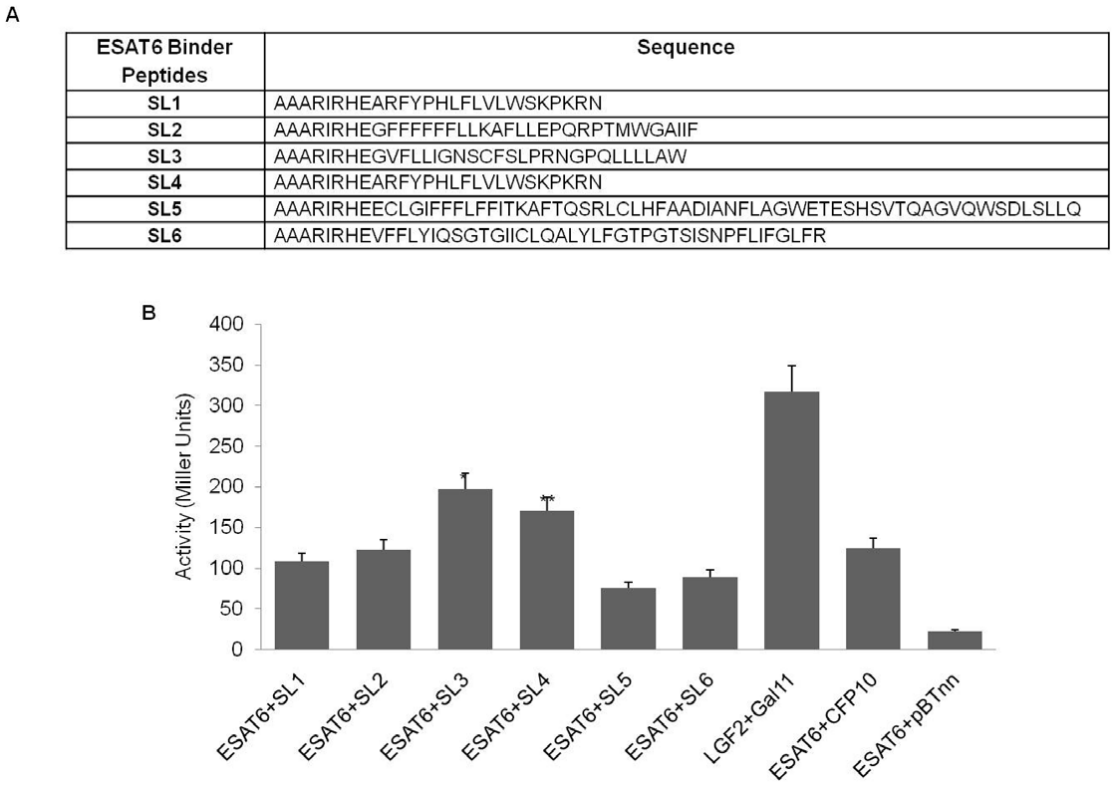
*De novo* ESAT6 binders. (A) Table showing peptide sequences of *de novo* ESAT6 binders. (B) Quantitative analysis of interacting strength of the binders (SL1-SL6) by liquid β-galactosidase assay. ESAT6 : CFP10 binding strength is shown as control; SL3 and SL4 show higher binding strength with ESAT6 compared to CFP10. Statistical significance was determined in comparison to the ESAT6 : CFP10 interaction (*, P<0.05; **, P<0.1).

To further test the stringency of our system, we wanted to investigate the behavior of CFP10 in the presence of a binder that bound ESAT6 more strongly than CFP10 itself. A bacterial two-hybrid screen was carried out using ESAT6 and the human lung cDNA library, cloned in pBTnn and pTRG vectors respectively. The experiment yielded a range of ESAT6 binders, named SL1-6. The binders were found to be small peptides having no homology with any known human protein (Fig 5A). On quantification of the strength of interaction, two peptides: SL3 and SL4, were observed to have a binding strength higher than ESAT6 : CFP10 (Fig 5B).

**Figure 6:**
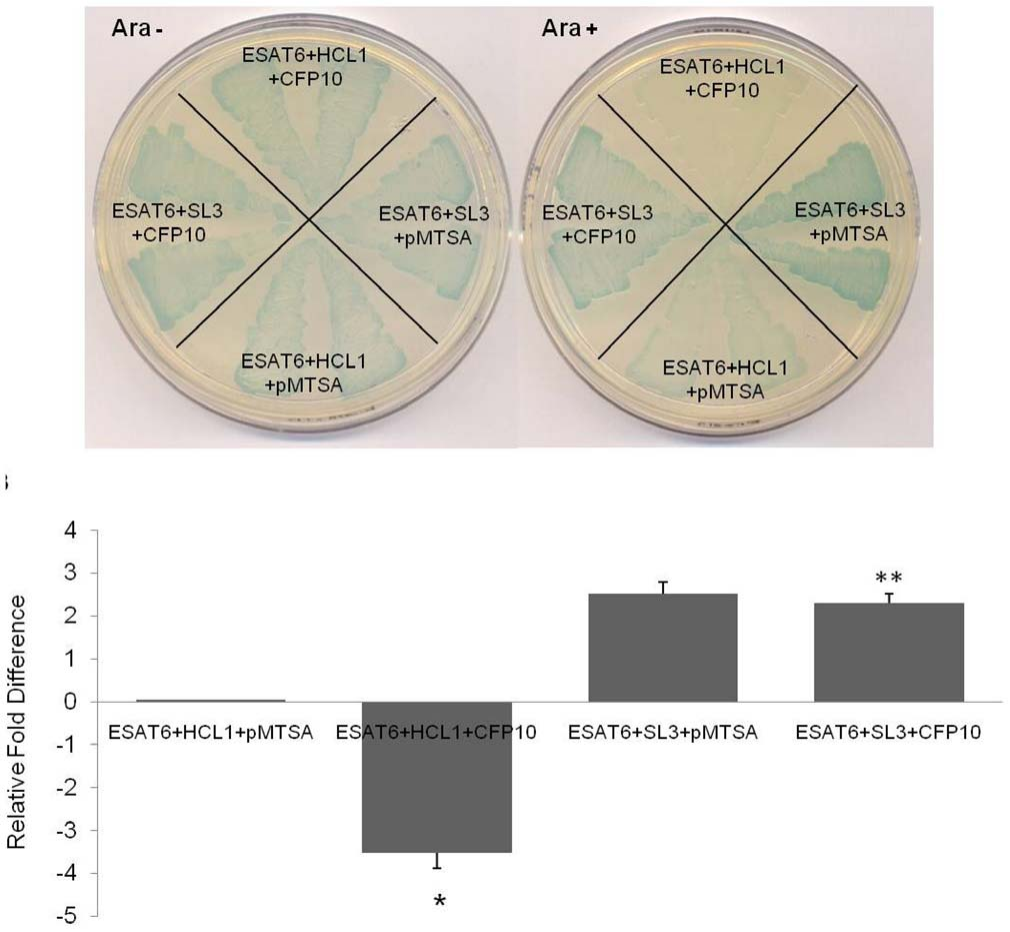
Representation of ESAT6 : CFP10 : SL3 interaction in bacterial three-hybrid system. (A) X-Gal indicator plates (with and without arabinose) showing triple co-transformants: ESAT6pBTnn + SL3pTRGnn + pMTSA; ESAT6pBTnn + HCL1pTRGnn + pMTSA; ESAT6pBTnn + SL3pTRGnn + CFP10pMTSA; and ESAT6pBTnn + HCL1pTRGnn + CFP10pMTSA. Two different colonies of each co-transformant were patched. (B) Fold-difference of *lacZ* gene transcript in the triple co-transformants relative to the ESAT6pBTnn + HCL1pTRGnn + pMTSA negative control strain. The *lacZ* gene transcription is reduced approx. four-fold as compared to negative control when ESAT6 and HCL1 interact in presence of CFP10 in the bacterial three-hybrid system (*, P<0.05), whereas no significant difference is observed in the ESAT6 : SL3 strain expressing CFP10 (**, P>0.05). The RT-PCR experiment was performed in triplicate using 3 separate colonies.

The stronger of the two binders, SL3, was investigated further using the bacterial three-hybrid assay system. The ‘blue reporter strain’ carrying ESAT6pBTnn and SL3pTRGnn was transformed with CFP10pMTSA (test) or pMTSA (control) plasmids and plated on arabinose positive and arabinose negative X-Gal indicator plates. Interestingly, presence or absence of CFP10 bore no effect, either on the visual intensity of blue color of the ESAT6 : SL3 strain (Fig 6A), or on its quantification by β-galactosidase assay (data not shown).

RT PCR analysis to quantify the *lacZ* gene transcription levels for both ESAT6 binders, HCL1 and SL3, was carried out. Indeed, the fold difference in the transcription levels for ESAT6 : HCL1, and ESAT6 : SL3, in presence and absence of CFP10, corroborated all our previous observations. A four-fold decrease was observed in the ESAT6 : HCL1 strain in the presence of CFP10 (in comparison with the negative control), while no significant difference for ESAT6 : SL3 reporter strain either in presence or in absence of CFP10 was observed (Fig 6B).

### Unearthing peptides that form tri-protein complexes

To investigate whether the three-hybrid system could be utilized to effectively unearth proteins or peptides that take part in a tri-protein complex, we decided to perform the following experiment: The ‘non-interacting’ reporter strain R1 was co-transformed with the plasmids CFP10pMTSA and ESAT6pBTnn and competent cells (mCER1) made. The mCER1 competent cells were transformed with an *M. tuberculosis* genomic DNA library (for the construction of the library please see Materials and Methods). Appearance of a blue-colored colony on plates that contained 1% Arabinose was an indication that the expression of a library member had facilitated the interaction between ESAT6 and CFP10. The pTRGnn-based library member was isolated both from a blue as well as a white colony, and used to retransform the mCER1 strain. Four colonies (labeled B1-4, Figure S7A) that appeared strong blue were picked from the initial library screening plate. Patching of these colonies along with the negative control was done on the Arabinose positive and Arabinose negative X-Gal Streptomycin plates. When observed for the development of blue color, only two (B1, B4) out of the initial four stayed blue on the Arabinose negative plate, while only B4 turned blue on the Arabinose positive plate. It can be inferred that in strain B1, the appearance of blue colored colony on Arabinose negative plate indicates that the pTRGnn library member interacted with ESAT6. Nonetheless, on Arabinose positive plate, the colony color stayed white, which signifies that the interaction was negated in the presence of CFP10 (the expression of CFP10 is regulated by presence or absence of Arabinose). However, in strain B4, the colony color was blue on both Arabinose positive and negative plates, indicating that the pTRGnn library member interacted with ESAT6 and the interaction was maintained even in the presence of CFP10.

The pTRGnn plasmids of each strain (B1 and B4) were segregated and used to re-transform the mCER1 compentent cells. The re-transformed strains were plated on Arabinose positive as well as Arabinose negative X-Gal Streptomycin plates. When allowed to develop at 30°C, both the strains (Reco B1 and Reco B4, Figure S7B) turned blue on the Arabinose negative plates, while only Reco B4 turned blue on the Arabinose positive plate. Sequencing of the pTRGnn plasmids B1 and B4 showed that both were small peptides (Figure S7C). B4 might therefore be a peptide candidate that forms a successful tri-protein complex with ESAT6 and CFP10. However, much more work needs to be done in this direction in order to fortify these initial results and carry out detailed analysis of the peptide and many other such proteins/peptides that form tri-protein complexes *in vivo*.

## Discussion

While a range of yeast two-hybrid and mycobacterial two-hybrid systems have been used for studying protein-protein interactions in *M. tuberculosis* [32]–[37], we believe that the use of an *E. coli* based bacterial system offers a few advantages, such as 1) ability to screen larger libraries 2) faster screening process 3) ability to screen eukaryotic as well as mycobacterial proteins which could be toxic or homologous to other proteins in the yeast and mycobacterial systems, and 4) a much more efficient induction of the proteins themselves.

Several modifications of yeast/bacterial two-hybrid systems in the form of yeast/bacterial three-hybrid systems have been reported in the past although most were applicable for RNAs and small molecules only [38]–[41]. A few groups have modified the yeast two-hybrid system to incorporate three different genes under regulation of different promoters to allow their independent expression and interaction [42]–[44]. Nevertheless, no such significant modification has been reported in a bacterial two-hybrid system except the one by Karimova and coworkers, which, although efficient, does not allow independent regulation of expression of the third gene [45].

In this report we have described a bacterial three-hybrid system that is a modification on the commercially available two-hybrid BacterioMatchI system [27], [28]. The *M. tuberculosis* EsxI system proteins have been used as a model for our studies.

To ascertain the utility of the system in studying tri-protein complexes, we applied the system to investigate the role of ESAT6 in the binding of CFP10 with Rv3871. Because ESAT6 and CFP10 form a tight 1∶1 heterodimeric complex, we initially used the standard two-hybrid system for studying the interaction of this complex with Rv3871, employing CFP10 and ESAT6 fusion constructs for this purpose. The results failed to throw more light on the vital role ESAT6 might play in the CFP10 : Rv3871 complex formation. On the other hand, using the three-hybrid system we were able to demonstrate conclusively the role of ESAT6. The results obtained on X-Gal Arabinose indicator plate (Fig 3A), liquid β-galactosidase assay (Fig 3B) and RT PCR (Fig 3C) showed that presence of ESAT6 strengthens the binding of CFP10 with Rv3871. It can be speculated from these observations that the binding of ESAT6 to CFP10 brings about certain conformational changes in the two proteins that then induces the exposure of the C-terminal tail of CFP10, ultimately making it available for stronger binding with Rv3871. Possibility of a direct intervention by ESAT6 in the binding of the complex with Rv3871 is also not ruled out, as indicated in a recent report by Callahan *et al* [46].

We also tested the suitability of the three-hybrid system for studying disruption of known protein-protein interactions by subjecting ESAT6 and its two known binder peptides: CFP10 and HCL1, to three-hybrid analysis. The observation on the X-Gal indicator plate and the quantitative analysis by liquid β-galactosidase assay clearly showed that the stronger of the two ESAT6 binders, CFP10, could dislodge the weaker binder HCL1. Here, the disruptor gene cloned in pMTSA vector is tightly regulated by the pBAD promoter allowing for its differential expression. Therefore, patching the test strain on arabinose positive and arabinose negative indicator plates enables a single step confirmation of the disruption of two interacting proteins by the third protein. Using the arabinose gradient liquid β-galactosidase assay we have shown the extent to which the level of expression of the pMTSA expressed gene (here CFP10) can be regulated and its effect exhibited on the protein-protein interaction. However, since HCL1 was a weaker binder of ESAT6 compared to CFP10, we next used a stronger binder, SL3, and investigated whether CFP10 could still dislodge SL3 from ESAT6. Absence of change in colony color on the arabinose positive and arabinose negative indicator plates confirmed the fact that CFP10 was unable to disrupt ESAT6 : SL3 interaction (Fig 6A).

Clearly, the strength of interaction between ESAT6 and both its non-natural binders, HCL1 and SL3, was different and affected its ability to bind its natural ligand CFP10. RT-PCR analysis showed that the strength of ESAT6 : SL3 interaction was approximately two-fold higher than the ESAT6 : HCL1 interaction (Fig 6B). Undoubtedly, while more work needs to be done to examine the effect of SL3 on the overall viability of *M. tuberculosis*, the three-hybrid system has indeed brought forth a binder, SL3, that binds to the well-established *M. tuberculosis* drug target ESAT6 with a binding strength higher than its natural ligand CFP10. Crucially, it is a binding that CFP10 itself cannot disrupt. This finding, we believe, has important implications for addressing inhibition of *M. tuberculosis* virulence and further studies would shed more light on the observed results.

That the three-hybrid approach could also be used to identify peptides that facilitate the formation of a tri-protein complex was also tested. A small peptide, B4, was seen to assist the formation of a tri-protein complex between itself, ESAT6, and CFP10. While these are initial studies and further work needs to be carried out to strengthen the basic approach, it is encouraging to note that the system offers discovery of small peptides that can bind to a known protein complex, like ESAT6 and CFP10, and go on to form a tri-protein complex. In *M. tuberculosis*, this application can be exploited in studying complex systems like the EsxI secretory system, where the interaction of proteins may occur only with dimeric complexes like CFP10:ESAT6 rather than with individual proteins. As dimerisation usually induces conformational changes in the proteins that have dimerised, a two hybrid system is not sufficient to study the interaction of a third protein with a member of the heterodimeric complex.

From our proof-of-concept studies, we understand that the bacterial three-hybrid system can be used not only for studying tri-protein complexes, but also for protein-protein disruption studies. For example, one could study the effect of a disruptor peptide (of say a particular protein-protein interaction) on the downstream biochemical pathway induced by the interaction itself. In the context of *M. tuberculosis*, this particular aspect could be vital for studying how this pathogen is able to evade the host machinery and propagate.

There is however a caveat in using a bacterial system for studying protein-protein interactions, especially when the interactions concern eukaryotic proteins: a prokaryotic codon-bias may result in low expression of the eukaryotic proteins; in some cases constituent library members of a eukaryotic cDNA library may even fail to express. One potential solution could be to allow for a more broad-based codon-usage in the two-hybrid bacterial strain that is used for the experiments. Additionally, for the eukaryotic proteins that are ultimately unearthed as potent binders and potential peptidomimetic templates for drugs against the target bacterial proteins, a re-synthesis of their corresponding genes keeping in mind a prokaryotic expression would permit further *E. coli*-based *in vitro* and *in vivo* studies. Nonetheless, the speed and ease with which eukaryotic protein/peptide entities can be revealed through the use of a bacterial system make it, we believe, a convenient strategy for discovering potent binders and inhibitors of target proteins.

## Materials and Methods

### DNA Sequencing

All plasmids described in this work were sequenced for the presence and integrity of their inserts using the Sanger method. No new information regarding DNA sequence (new gene, organism, etc.) was generated in the course of this work that merited submission to GenBank.

### Bacterial Two-Hybrid Studies

The bacterial two-hybrid studies were carried out using Bacteriomatch™ two-hybrid system vector kit (Stratagene, USA). The kit was supplied with bacterial two-hybrid system plasmids pBT, pTRG and control plasmids pBT-LGF2 and pTRG-Gal11p. The pBT and pTRG vectors were modified to have a unique *Sna*BI site in MCS for blunt end clonings and renamed pBTnn and pTRGnn respectively [31]. The human lung cDNA library (cloned in pTRG vector) was purchased from Stratagene, USA. Full length genes for *esat6* (Rv3875), *cfp10* (Rv3874) and *Rv3871* were amplified from *M. tuberculosis* (H37Rv) genomic DNA using their respective forward and reverse primers (Table 1). The PCR products were digested with *Sna*BI and cloned in *Sna*BI*-*cut pTRGnn and pBTnn vectors. A fusion of CFP10 and ESAT6 proteins (*cfp10-esat6*) was constructed where *esat6* was fused to the C-terminal of *cfp10* gene. The CFP10pBTnn clone was digested with *Sca*I at a site located immediately upstream of C-terminal end of *cfp10* gene to provide free blunt ends. *Sna*BI*-*digested insert of *esat6* gene was cloned at this site to yield the CFP10-ESAT6pBTnn clone. Another fusion construct where *esat6* was fused at the N-terminal of *cfp10* gene (*esat6-cfp10*), was made by cloning a *Sna*BI*/Sca*I digested *cfp10* insert in *Sna*BI-cut pBTnn vector backbone. *Esat6* insert was then cloned in the *Sna*BI*-*digested construct to give ESAT6-CFP10pBTnn. The genes coding for HCL1 and SL3 peptides were obtained from human lung cDNA library and recloned in pTRGnn vector using gene-specific forward and reverse primers as described earlier [31]. Reporter strain competent cells were co-transformed with equal amount (250 ng each) of the respective pBTnn and pTRGnn clones and plated on X-Gal plates containing: kanamycin (50 µg/mL), chloramphenicol (30 µg/mL), tetracycline (12.5 µg/mL), X-Gal (80 µg/mL), IPTG (25 µM), and X-Gal inhibitor (phenylmethyl-β-D-thiogalactoside) (200 µM). Plasmids pBT-LGF2 (λcI-LGF2 fusion) and pTRG-Gal11p (RNAPα subunit–Gal11p fusion) provided the positive controls, whereas empty pBTnn and pTRGnn plasmids served as negative controls for *in vivo* interaction studies. Positive interactions were judged by the blue color of the colonies obtained and verified by repeated sub-clonings and co-transformations. The interactions were further confirmed by performing liquid β-galactosidase assay as described earlier [47]. The significance of each interaction was determined by statistical analysis using Student's t-test.

**Table 1:** The sequence of gene specific forward and reverse primers used in this work.

CFP10	Forward	5′-CCTACGTAATGGCAGAGATGAAGACCGA-3′
	Reverse	5′-CCTACGTAAGTACTGAAGCCCATTTGCGAGGACA-3′
ESAT6	Forward	5′-CCTACGTAATGACAGAGCAGCAGTGGAA-3′
	Reverse	5′-CCTACGTATGCGAACATCCCAGTGACGT-3′
Rv3871	Forward	5′-CCTACGTAATGACTGCTGAACCGGAAGTACGGAC-3′
	Reverse	5′-CCTACGTAACCGGCGCTTGGGGGTGCTGCGAAC-3′
HCL1	Forward	5′-AAGGATCCTACGTAAGAATTCGGCACGAG-3′
	Reverse	5′-AAGGATCCTACGTAATAATCATTAAACTT-3′
SL3	Forward	5′-AAGGATCCTACGTAAGAATTCGGCACGAG-3′
	Reverse	5′-AAGGATCCTACGTACCAGGCGAGCAGGAGC -3′

### Construction of pMTSA vector

The original streptomycin-resistant pCDFDuet vector (Clonetech, USA) was modified to have an AraC ORF (open reading frame) and an MCS carrying *Sna*B1 restriction site under the strong pBAD promoter. A DNA fragment of size 2306 bp was extracted from the original pBADHisAnn vector [31] by digesting with restriction enzymes *Xmn*I and *Nde*I. This fragment was cloned in *Nde*I*/Pvu*II-digested pCDFDuet vector (2299 bp fragment). *E. coli* DH5α competent cells were transformed with the ligation product and plated on LB-streptomycin plate. Positive clones were confirmed by restriction digestion and reconfirmed by sequencing using pBAD forward primer. The resulting expression vector was designated as pMTSA which now had a CDF origin of replication (for compatibility and high copy number), an arabinose inducible pBAD promoter, and an MCS (multiple cloning site) containing a unique *Sna*BI restriction site to facilitate blunt end clonings.

### Cloning and expression of hcl1, esat6 and cfp10 in pMTSA vector

ORFs corresponding to these genes were PCR-amplified using their respective forward and reverse primers (Table 1) followed by *Sna*B1 digestion of the PCR products. Subsequent inserts were cloned in *Sna*B1-digested pMTSA vector to give HCL1pMTSA, ESAT6pMTSA and CFP10pMTSA clones respectively. Expression of proteins in pMTSA clones was checked by transforming *E. coli* BL21(DE3) cells and inducing the bacterial culture with 0.2% arabinose in the presence of streptomycin (50 mg/mL). Whole cell lysates were run on SDS-PAGE and stained with Coomassie Brilliant Blue R-250 stain. All proteins were expressed as fusion proteins with 6x His tag at their C-terminal end.

### Bacterial three-hybrid studies

Reporter competent cells were transformed with the pTRGnn and pBTnn vectors carrying the genes corresponding to the interacting proteins and allowed to grow on X-Gal plates. Competent cells were prepared from the blue strain and transformed with pMTSA plasmid encoding the third interacting protein and grown on X-Gal arabinose indicator plate containing: kanamycin (50 µg/mL), chloramphenicol (30 µg/mL), tetracycline (12.5 µg/mL), streptomycin (50 µg/mL), X-Gal (80 µg/mL), IPTG (25 µM), L-arabinose (1%), and X-Gal inhibitor (200 µM). The blue strain carrying empty pMTSA vector was taken as the negative control. Colonies were grown at 30°C and observed for development of blue color. Effect of pMTSA-expressed protein on the interacting proteins was judged by the change in intensity of blue color of the colony as compared to the negative control.

### Time Course Liquid β-galactosidase Assay

The selected triple co-transformant and the corresponding negative control were grown to mid-log phase in presence of 40 µM IPTG. Subsequent induction with 1% arabinose was carried out. The induced cultures were allowed to grow at 37°C with shaking. 500 µL of each culture sample was collected at regular intervals from the time of induction till a further 3 hours. Culture pellet of the samples of each time point was taken and colorimetric liquid β-galactosidase assay using ONPG (2-Nitrophenyl β-D-galactopyranoside) as substrate was carried out as described earlier [47]. Statistical analysis was carried out by using Student's t-test.

### Arabinose Gradient Liquid β-galactosidase Assay

The selected triple co-transformant and the corresponding negative control were grown to mid-log phase in the presence of 40 µM IPTG. Induction was done with varying arabinose concentrations ranging from 0 to 1%. Growth of induced culture was allowed for a further 3 hours. 500 µL culture pellet of each sample was taken and assayed colorimetrically by liquid β-galactosidase assay as described elsewhere [47]. Student's t-test was used for the statistical analysis.

### Screening of tri-protein complexes

R1 reporter strain was co-transformed with the plasmids CFP10pMTSA and ESAT6pBTnn, and plated on LB plate containing Streptomycin (50 µg/ml) and Chloramphenicol (30 µg/ml). A single colony was isolated and taken to prepare chemical competent cells (mCER1). The mCER1 competent cells were transformed with an *M. tuberculosis* genomic DNA library contained in the two-hybrid plasmid pTRGnn. The transformed cells were plated on X-Gal Streptomycin plate containing 1% Arabinose and allowed to grow at 30°C for about 40 hrs. The blue colonies observed on the plates were isolated and grown overnight in a liquid culture. 10 µl of each culture was taken and patched on the Arabinose positive and Arabinose negative X-Gal Streptomycin plates. A negative control carrying empty pTRG plasmid in the mCER1 competent cells was also patched on the same plates. The plates were incubated and observed for the development of blue color. The colonies that had turned blue on the Arabinose negative X-gal Streptomycin plate were taken for further analysis. Plasmid isolation was carried out by mini-prep and checked by PCR for the presence of all three plasmids (ESAT6pBTnn, CFP10pMTSA and the pTRGnn plasmid carrying the library member). Segregation of the pTRGnn plasmid of each isolate was then carried out by transforming R1 competent cells with the plasmid mix and plating them on LB Agar plates containing only tetracycline (12.5 µg/ml), the resistance marker present on the pTRG plasmid. Five colonies were picked from each tetracycline plate and checked by PCR to confirm the absence of ESAT6pBTnn and CFP10pMTSA plasmids. The PCR-confirmed colonies from each isolate were picked and taken for plasmid isolation. The pTRGnn plasmid of each isolate was taken to retransform the mCER1 competent cells and allowed to grow on Arabinose positive and Arabinose negative X-Gal plates till the appearance of blue color. The pTRGnn plasmids were further sent for sequence analysis.

### Construction of M. tuberculosis genomic DNA library

*M. tuberculosis* H37Rv Genomic DNA was subjected to partial digestion with restriction enzyme *Hae*III. The digested genome was run on a 1% agarose gel and of the resulting numerous fragments, those ranging between 500 bp and 3 kb were eluted. After these fragments were purified, ‘hairpins’ (HP) were ligated to either side of these fragments [26]. Subsequently, these hairpin ligated fragments were digested with *Xba*I and then amplified by PCR using phosphorylated HP primers, in order to facilitate blunt ended ligations into *Sna*BI cut vector pTRGnn.

### Cloning, Expression and Purification of proteins

To obtain GST-tagged CFP10 protein, BL21(DE3) cells were transformed with *cfp10* gene cloned in pGex4T3nn [31] and grown in liquid culture till mid-log phase. The culture was induced with 1 mM IPTG and grown for 3 hours at 37°C with shaking. Cells were harvested and lysed. Lysate was treated with 1% Triton X-100 and centrifuged. The supernatant was incubated with washed and equilibrated glutathione-agarose beads (Amersham) with constant shaking at 4°C for 5 hours. Elution of the CFP10-GST protein from the beads was carried out using elution buffer (10 mM reduced Glutathione in 10 mM Tris-Cl, pH 8.0). Eluates were collected and the integrity of protein was checked on SDS-PAGE. Quantification of protein was done by Bradford assay.

*Rv3871* gene was PCR-amplified from the *M. tuberculosis* genomic DNA using gene specific primers (Table 1) and cloned in the pBADHisAnn [31] vector. To obtain His-tagged Rv3871 protein, BL21(DE3) cells harboring Rv3871pBADHisAnn plasmid were grown till mid-log phase and induced with 0.02% arabinose. Induced cells were grown for 3 hours with shaking at 37°C and harvested followed by their lysis. Supernatant was incubated with washed and equilibrated Ni-NTA agarose beads with shaking for 5 hours at RT. Elution was carried out with Tris-NaCl buffer containing 250 mM Imidazole. Eluates were collected and checked on SDS-PAGE. Purified protein was refolded sequentially by slowly dialyzing against Tris-NaCl buffer (20 mM Tris, 150 mM NaCl) with decreasing concentration of urea. Final dialysis in 0 M Urea was carried out in presence of 50 mM L-Arginine. Protein was concentrated using Amicon Ultra Millipore column.

Purification of HCL1-GST and ESAT6-His proteins was achieved by the same protocol as described earlier [31].

### Far-Western Dot Blot Assay

1 µg of His-tagged purified protein was immobilized as a small blot on nitrocellulose membrane and allowed to dry. Membrane was blocked with 5% skimmed milk [in PBS (Phosphate buffer saline) containing 0.1% Tween] for two hours and washed with 0.1% PBST. The membrane immobilized protein was incubated overnight with 1 µg/mL solution of GST-tagged binder protein (in 2.5% skimmed milk in 0.1% PBST solution) at 4°C with shaking. Membrane was again washed with 0.1% PBST and incubated with anti-GST antibodies (Sigma) (1∶2000) for 2 hours. Finally, incubation with anti-mouse IgG HRPO-labeled secondary antibodies (Calbiochem) (1∶5000) was carried out for 2 hours. The blot was developed using TMB (3,3′,5,5′-tetramethylbenzidine) substrate (Sigma).

### Quantitative Real Time PCR studies

The selected triple co-transformant and the corresponding negative control were grown to mid-log phase. Each culture was subsequently induced with 1% arabinose and allowed to grow for 3 hours. 500 µL of culture was pelleted down and total RNA was isolated as described previously [31]. *DNase*I (Fermentas, USA) treatment was given as per manufacturer's instructions. Subsequent purification was carried out with RNeasy mini prep kit (Qiagen) column according to the manufacturer's protocol. RNA was checked for integrity and purity by running on 1% denaturing agarose gel. Quantity and quality check of the RNA was done using NanoDrop ND-1000 spectrophotometer (Thermo Scientific). 1 µg of this RNA was reverse-transcribed to cDNA using BioRad iScript cDNA Synthesis Kit as described by the manufacturer. Real-time PCR was performed with 1 µL of diluted cDNA template (equivalent to 8 ng). *LacZ* forward (5′-ATG ACC ATG ATT ACG GAT TC-3′) and reverse primers (5′-ATT CGC CAT TCA GGC TGC GCA-3′) and IQ SYBR Green Supermix (BioRad) were used to prepare the PCR mix. Amplification was performed in a MiniOpticon (BioRad) Thermocycler using the protocol described earlier [31]. The gene coding for kanamycin resistance was used for the normalization of comparative C_t_ values. ‘No template’ controls (no cDNA in PCR) were run for each gene to check for non-specific amplification or primer dimerization. Statistical significance of each reading was calculated using Student's t-test.

## Supporting Information

Figure S1 **Expression of Rv3871 gene in pMTSA vector in reporter strain R1.** The ability of pMTSA vector to express the cloned insert in the bacterial two-hybrid Reporter strain R1 was checked by cloning Rv3871 gene in the pMTSA vector and allowing for expression under arabinose induction. Lane 1: Protein Ladder (BioRad) Molecular weight in kDa; Lane 2: Uninduced cell lysate; Lane 3: 0.2% arabinose induced cell lysate expressing the 65 kDa Rv3871 protein.
(TIF)

Figure S2 **In vitro representation of protein-protein interaction of CFP10 with ESAT6 and Rv3871.** (A) 15% SDS-PAGE stained with coomassie blue showing purified proteins. Lane M: Protein Ladder (BioRad) Molecular weight in kDa; Lane 1: Rv3871-His protein (65 kDa); Lane 2: CFP10-GST protein (36 kDa); Lane 3: HCL1-GST protein (28 kDa); Lane 4: GST protein (26 kDa); Lane 5: ESAT6-His protein (10 kDa). (B) Far Western Dot Blot Assay: 1 µg each of purified CFP10-GST protein and purified GST protein (negative control) were blotted on two separate strips of nitrocellulose membranes and incubated with 1 µg/mL solution of purified ESAT6-His or Rv3871-His. Blots were developed using anti-His antibody. (C) Spot Densitometric Analysis for the quantitative estimation of the blots obtained by Far Western Dot Blot confirmed a strong interaction between CFP10 and ESAT6, and weaker interaction between CFP10 and Rv3871.
(TIF)

Figure S3 **Representation of protein-protein interaction of the CFP10 and ESAT6 fusion constructs with Rv3871 in bacterial two-hybrid system.** (A) Bacterial two-hybrid X-Gal plate showing co-transformants CFP10pBTnn + Rv3871pTRGnn; CFP10-ESAT6pBTnn + Rv3871pTRGnn; ESAT6-CFP10pBTnn + Rv3871pTRGnn; and pBTnn + Rv3871pTRGnn (negative control). Two different colonies of each co-transformant were patched (B) Quantitative analysis by liquid β-galactosidase assay. The graph is the average of three separate assays and standard deviation is represented as error bars. (*, P<0.02; **, P<0.05; ***, P<0.01).
(TIF)

Figure S4 **RT-PCR analysis to confirm equivalent expression of CFP10 and Rv3871 in the ESAT6 positive and negative three-hybrid strains.** No difference in the transcription level of CFP10 and Rv3871 was observed in the three-hybrid strains CFP10pBTnn+Rv3871pTRGnn+ESAT6pMTSA and CFP10pBTnn +Rv3871pTRGnn+pMTSA indicating that the gradation in the colony blue color of the two strains was solely due to the differential interaction of CFP10 and Rv3871 in the strains influenced by the presence or absence of ESAT6. Kanamycin was used as the internal control. The graph shows an average of three separate assays.
(TIF)

Figure S5 **ESAT6 : HCL1 protein-protein interaction.** (A) Bacterial two-hybrid X-Gal plate patched with two separate colonies each, of co-transformants LGF2pBTnn + Gal11pTRGnn (positive control); ESAT6pBTnn + CFP10pTRGnn; ESAT6pBTnn + HCL1pTRGnn; and pBTnn + HCL1pTRGnn (negative control) (B) Quantitative representation by liquid β-galactosidase assay. The graph is the average of three independent assays and standard deviation is represented as error bars. (*, P<0.005; **, P<0.02) (C) Far Western Dot Blot Assay: 1 µg each of purified proteins ESAT6-His, CFP10-His (negative control), and GST (positive control) were spotted on nitrocellulose membrane and incubated with 1 µg/mL solution of purified HCL1-GST protein. Blot was developed using anti-GST antibodies.
(TIF)

Figure S6 **Representation of disruption of ESAT6 : HCL1 protein-protein interaction by CFP10 in bacterial three-hybrid system.** (A) X-Gal indicator plate with and without arabinose patched with ESAT6pBTnn + HCL1pTRGnn + HLL7pMTSA; and ESAT6pBTnn + HCL1pTRGnn + CFP10pMTSA. Blue colony turns white when CFP10 is allowed to express in the presence of 1% arabinose while no effect on colony color on expression of the dummy non-interacting peptide HLL7 is seen (B) Time course liquid β-galactosidase assay: β-galactosidase activity of the triple co-transformants: (▴) ESAT6pBTnn + HCL1pTRGnn + HLL7pMTSA; and (▪) ESAT6pBTnn + HCL1pTRGnn + CFP10pMTSA is plotted against time-points of bacterial culture growth with 0 time-point being the point of arabinose induction. Standard deviation of the activities obtained in three separate assays is shown by error bars. (P<0.01 at all time-points beyond 90 minutes).
(TIF)

Figure S7 **Discovery of a peptide that facilitates the formation of a tri-protein complex.** (A) Patching of colonies B1-4 on Arabinose positive and negative plates. B4 remains blue on both plates. (B) Re-cotransformation of mCER1 competent cells with pTRGnn-based library members B1 and B4. RecoB4 remains blue on both Arabinose negative as well as on Arabinose positive plates. (C) Peptide sequences of B1 and B4.
(TIF)

## References

[1] WHO Media centre (2010). http://www.who.int/mediacentre/factsheets/fs104/en/index.html.

[2] Suchindran S, Brouwer ES, Van Rie A (2009). Is HIV infection a risk factor for multi-drug resistant tuberculosis? A systematic review.. PLoS One.

[3] Wells CD, Cegielski JP, Nelson LJ, Laserson KF, Holtz TH (2007). HIV infection and multidrug-resistant tuberculosis: the perfect storm.. J Infect Dis.

[4] Chao MC, Rubin EJ (2010). Letting Sleeping dos Lie : Does Dormancy Play a Role in Tuberculosis?. Annu Rev Microbiol.

[5] Cosma CL, Sherman DR, Ramakrishnan L (2003). The secret lives of the pathogenic mycobacteria.. Annu Rev Microbiol.

[6] Davis JM, Ramakrishnan L (2009). The role of the granuloma in expansion and dissemination of early tuberculous infection.. Cell.

[7] Cui T, Zhang L, Wang X, He Z (2009). Uncovering new signaling proteins and potential drug targets through the interactome analysis of Mycobacterium tuberculosis.. BMC Genomics.

[8] Chao J, Wong D, Zheng X, Poirier V, Bach H (2010). Protein kinase and phosphatase signaling in Mycobacterium tuberculosis physiology and pathogenesis.. Biochim Biophys Acta.

[9] Dietrich J, Doherty TM (2009). Interaction of Mycobacterium tuberculosis with the host: consequences for vaccine development.. APMIS.

[10] Vergne I, Chua J, Lee H, Lucas M, Belisle J (2005). Mechanism of phagolysosome biogenesis block by viable Mycobacterium tuberculosis.. Proc Natl Acad Sci USA.

[11] Bach H, Papavinasasundaram KG, Wong D, Hmama Z, Av-gay Y (2008). Mycobacterium tuberculosis virulence is mediated by PtpA dephosphorylation of human vacuolar protein sorting 33B.. Cell Host Microbe.

[12] Renshaw PS, Panagiotidou P, Whelan A, Gordon SV, Hewinson RG (2002). Conclusive evidence that the major T-cell antigens of the Mycobacterium tuberculosis complex ESAT-6 and CFP-10 form a tight, 1∶1 complex and characterization of the structural properties of ESAT-6, CFP-10, and the ESAT-6*CFP-10 complex. Implications for pathogenesis and virulence.. J Biol Chem.

[13] Guinn KM, Hickey MJ, Mathur SK, Zakel KL, Grotzke JE (2004). Individual RD1-region genes are required for export of ESAT-6/CFP-10 and for virulence of Mycobacterium tuberculosis.. Mol Microbiol.

[14] Champion PA, Stanley SA, Champion MM, Brown EJ, Cox JS (2006). C-terminal signal sequence promotes virulence factor secretion in Mycobacterium tuberculosis.. Science.

[15] Champion PA, Champion MM, Manzanillo P, Cox JS (2009). ESX-1 secreted virulence factors are recognized by multiple cytosolic AAA ATPases in pathogenic mycobacteria.. Mol Microbiol.

[16] Champion PA, Champion MM, Manzanillo P, Cox JS (2009). ESX-1 secreted virulence factors are recognized by multiple cytosolic AAA ATPases in pathogenic mycobacteria.. Mol Microbiol.

[17] Pym AS, Brodin P, Majlessi L, Rosch R, Demangel C (2003). Recombinant BCG exporting ESAT-6 confers enhanced protection against tuberculosis.. Nat Med.

[18] Stanley SA, Raghavan S, Hwang WW, Cox JS (2003). Acute infection and macrophage subversion by Mycobacterium tuberculosis require a specialized secretion system.. Proc Natl Acad Sci USA.

[19] Hsu T, Hingley-Wilson S, Chen B, Chen M, Dai A et (2003). The primary mechanism of attenuation of bacillus Calmette-Guerin is a loss of secreted lytic function required for invasion of lung interstitial tissue.. Proc Natl Acad Sci USA.

[20] Volkman HE, Clay H, Beery D, Chang JC, Sherman DR (2004). Tuberculous granuloma formation is enhanced by a mycobacterium virulence determinant.. PLoS Biol.

[21] Volkman H, Pozos T, Zheng J, Davis J, Rawls J (2010). Tuberculous granuloma induction via interaction of a bacterial secreted protein with host epithelium.. Science.

[22] Fletcher S, Hamilton AD (2007). Protein–Protein interaction inhibitors: small molecules from screening techniques.. Curr Topics Med Chem.

[23] Loregian A, Palu G (2005). Disruption of protein–protein interactions: towards new targets for chemotherapy.. J Cell Physiol.

[24] Sperandio O, Miteva MA, Segers K, Nicolaes GA, Villoutreix BO (2008). Screening Outside the Catalytic Site: Inhibition of Macromolecular Inter-actions Through Structure-Based Virtual Ligand Screening Experiments.. Open Biochem J.

[25] Chopra S, Ranganathan A (2003). Protein evolution by "codon shuffling": a novel method for generating highly variant mutant libraries by assembly of hexamer DNA duplexes.. Chem Biol.

[26] Rao A, Chopra S, Ram G, Gupta A, Ranganathan A (2005). Application of the "codon-shuffling" method. Synthesis and selection of de novo proteins as antibacterials.. J Biol Chem.

[27] Dove SL, Joung JK, Hochschild A (1997). Activation of prokaryotic transcription through arbitrary protein–protein contacts.. Lett Nat.

[28] Dove SL, Hochschild A (1998). Conversion of the omega subunit of Escherichia coli RNA polymerase into a transcriptional activator or an activation target.. Genes Dev.

[29] Meher AK, Bal NC, Chary KV, Arora A (2006). Mycobacterium tuberculosis H37Rv ESAT-6-CFP-10 complex formation confers thermodynamic and biochemical stability.. FEBS J.

[30] Renshaw PS, Lightbody KL, Veverka V, Muskett FW, Kelly G (2005). Structure and function of the complex formed by the tuberculosis virulence factors CFP-10 and ESAT-6.. EMBO J.

[31] Kumar K, Tharad M, Ganapathy S, Ram G, Narayan A (2009). Phenylalanine-rich peptides potently bind ESAT6, a virulence determinant of Mycobacterium tuberculosis, and concurrently affect the pathogen's growth.. PLoS One.

[32] Parida BK, Douglas T, Nino C, Dhandayuthapani S (2005). Interactions of anti-sigma factor antagonists of Mycobacterium tuberculosis in the yeast two-hybrid system.. Tuberculosis (Edinb.).

[33] Curry JM, Whalan R, Hunt DM, Gohil K, Strom M (2005). An ABC transporter containing a forkhead-associated domain interacts with a serine-threonine protein kinase and is required for growth of Mycobacterium tuberculosis in mice.. Infect Immun.

[34] Garg S, Alam S, Bajpai R, Kishan KV, Agrawal P (2009). Redox biology of Mycobacterium tuberculosis H37Rv: protein-protein interaction between GlgB and WhiB1 involves exchange of thiol-disulfide.. BMC Biochem.

[35] Singh A, Mai D, Kumar A, Steyn AJ (2006). Dissecting virulence pathways of Mycobacterium tuberculosis through protein-protein association.. Proc Natl Acad Sci USA.

[36] O'Hare H, Juillerat A, Dianišková P, Johnsson K (2008). A split-protein sensor for studying protein-protein interaction in mycobacteria.. J Microbiol Methods.

[37] Chang Y, Mead D, Dhodda V, Brumm P, Fox BG (2009). One-plasmid tunable coexpression for mycobacterial protein-protein interaction studies.. Protein Sci.

[38] Althoff EA, Cornish VW (2002). A bacterial small-molecule three-hybrid system.. Angew Chem Int Ed.

[39] Firestine SM, Salinas F, Nixon AE, Baker SJ, Benkovic SJ (2000). Using an AraC-based three-hybrid system to detect biocatalysts in vivo.. Nat Biotechnol.

[40] Licitra JE, Liu JO (1996). A three-hybrid system for detecting small ligand–protein receptor interactions.. Proc Natl Acad Sci USA.

[41] Sengupta DJ, Zhangt B, Kraemert B, Pochart P, Fields S (1996). A three-hybrid system to detect RNA-protein interactions in vivo.. Proc Natl Acad Sci USA.

[42] Vidal M, Brachmann RK, Fattaey A, Harlow E, Boeke JD (1996). Reverse two-hybrid and one-hybrid systems to detect dissociation of protein-protein and DNA-protein interactions.. Proc Natl Acad Sci USA.

[43] Tirode F, Malaguti C, Romero F, Attar R, Camonis J (1997). A conditionally expressed third partner stabilizes or prevents the formation of a transcriptional activator in a three-hybrid system.. J Biol Chem.

[44] Zhang J, Lautar S (1996). A yeast three-hybrid method to clone ternary protein complex components.. Anal Biochem.

[45] Karimova G, Dautin N, Ladant D (2005). Interaction network among Escherichia coli membrane proteins involved in cell division as revealed by bacterial two-hybrid analysis.. J Bacteriol.

[46] Callahan B, Nguyen K, Collins A, Valdes K, Caplow M (2010). Conservation of structure and protein-protein interactions mediated by the secreted mycobacterial proteins EsxA, EsxB, and EspA.. J Bacteriol.

[47] Miller JH (1972). Experiments in molecular genetics..

