# Supplementary figures and images for: A Three-Hybrid System to Probe In Vivo Protein-Protein Interactions: Application to the Essential Proteins of the RD1 Complex of M. tuberculosis
Source: PLoS One. 2011 Nov 8;6(11):e27503. doi: 10.1371/journal.pone.0027503 (PMC3210800; doi:10.1371/journal.pone.0027503)

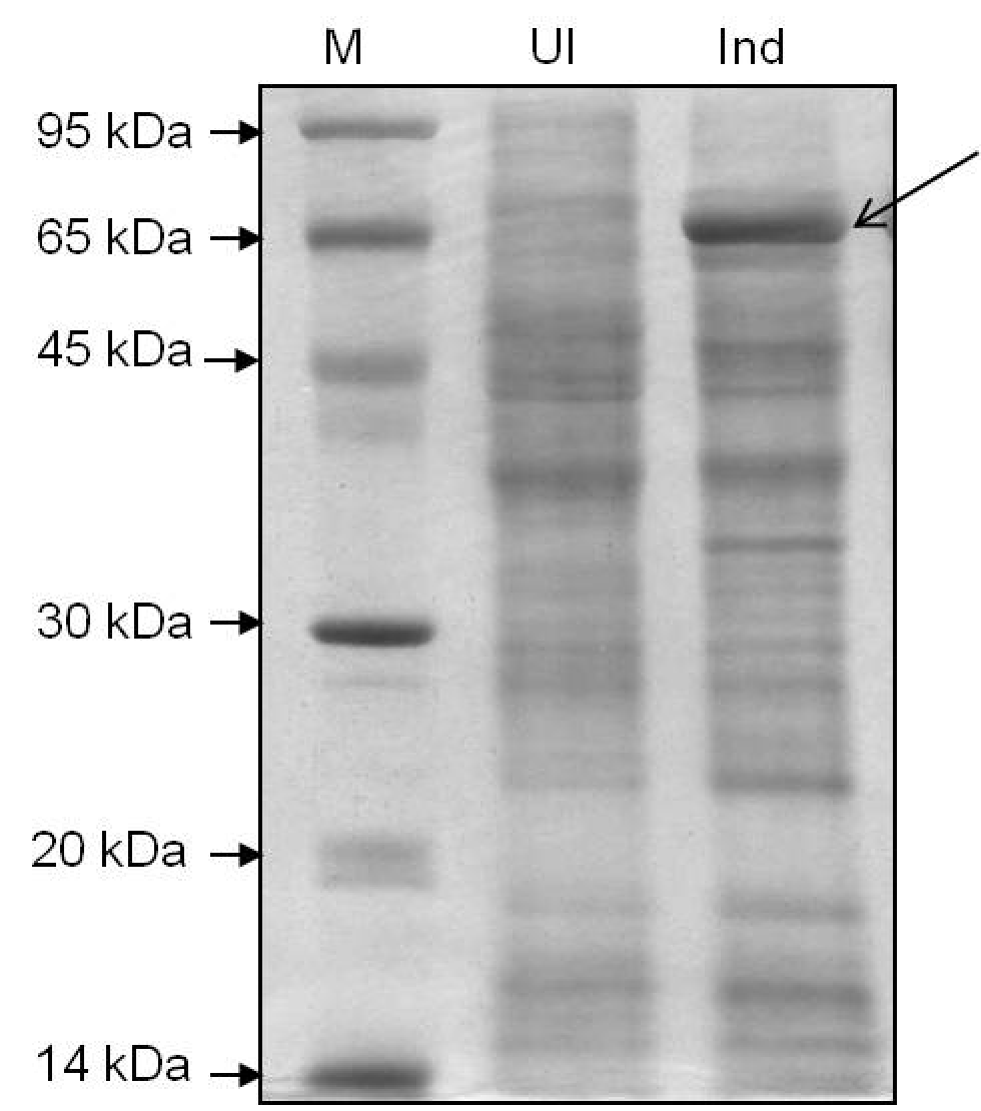

Supplement: Figure S1 — Expression of Rv3871 gene in pMTSA vector in reporter strain R1. The ability of pMTSA vector to express the cloned insert in the bacterial two-hybrid Reporter strain R1 was checked by cloning Rv3871 gene in the pMTSA vector and allowing for expression under arabinose induction. Lane 1: Protein Ladder (BioRad) Molecular weight in kDa; Lane 2: Uninduced cell lysate; Lane 3: 0.2% arabinose induced cell lysate expressing the 65 kDa Rv3871 protein. (TIF) [file pone.0027503.s001.tif]

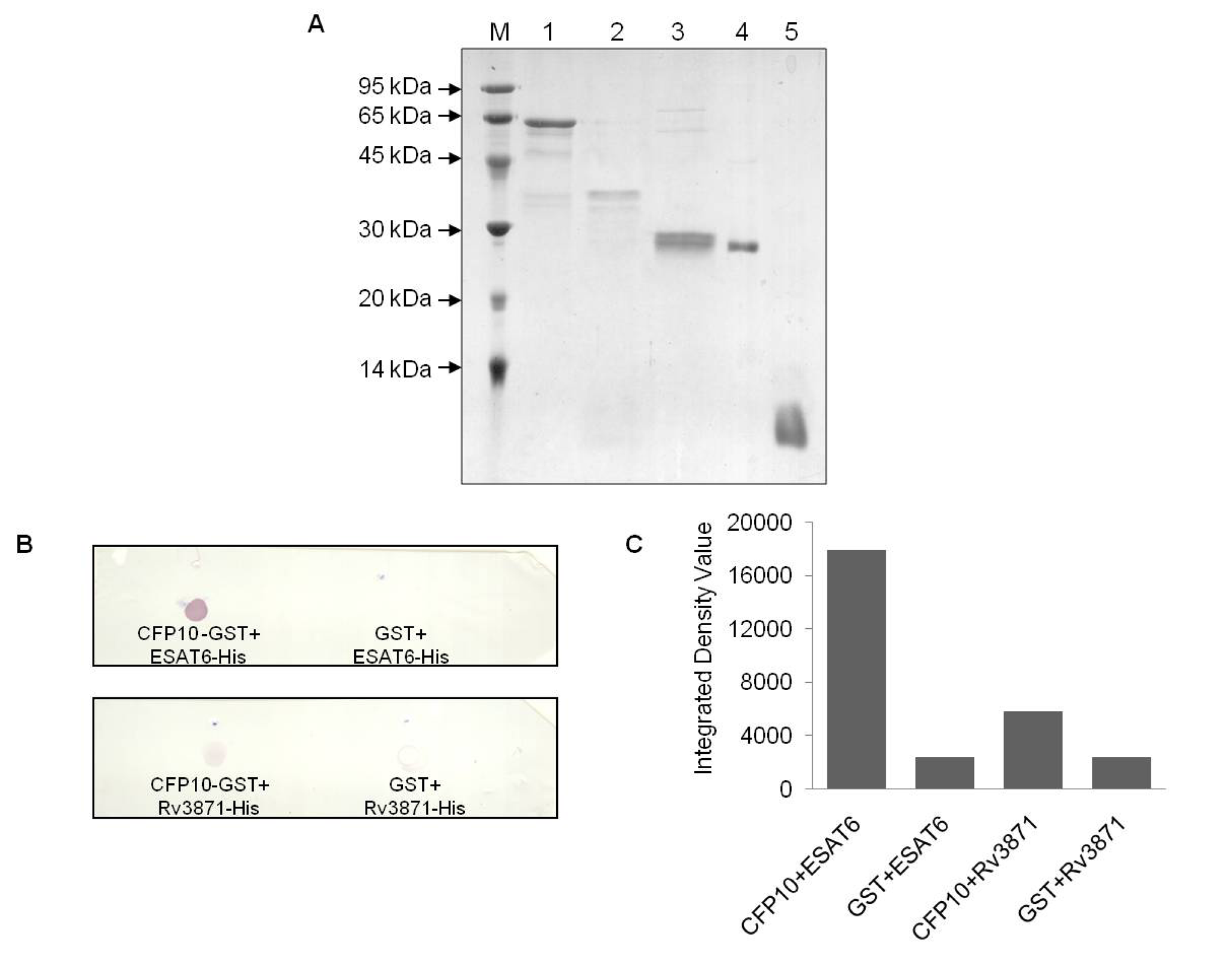

Supplement: Figure S2 — In vitro representation of protein-protein interaction of CFP10 with ESAT6 and Rv3871. (A) 15% SDS-PAGE stained with coomassie blue showing purified proteins. Lane M: Protein Ladder (BioRad) Molecular weight in kDa; Lane 1: Rv3871-His protein (65 kDa); Lane 2: CFP10-GST protein (36 kDa); Lane 3: HCL1-GST protein (28 kDa); Lane 4: GST protein (26 kDa); Lane 5: ESAT6-His protein (10 kDa). (B) Far Western Dot Blot Assay: 1 µg each of purified CFP10-GST protein and purified GST protein (negative control) were blotted on two separate strips of nitrocellulose membranes and incubated with 1 µg/mL solution of purified ESAT6-His or Rv3871-His. Blots were developed using anti-His antibody. (C) Spot Densitometric Analysis for the quantitative estimation of the blots obtained by Far Western Dot Blot confirmed a strong interaction between CFP10 and ESAT6, and weaker interaction between CFP10 and Rv3871. (TIF) [file pone.0027503.s002.tif]

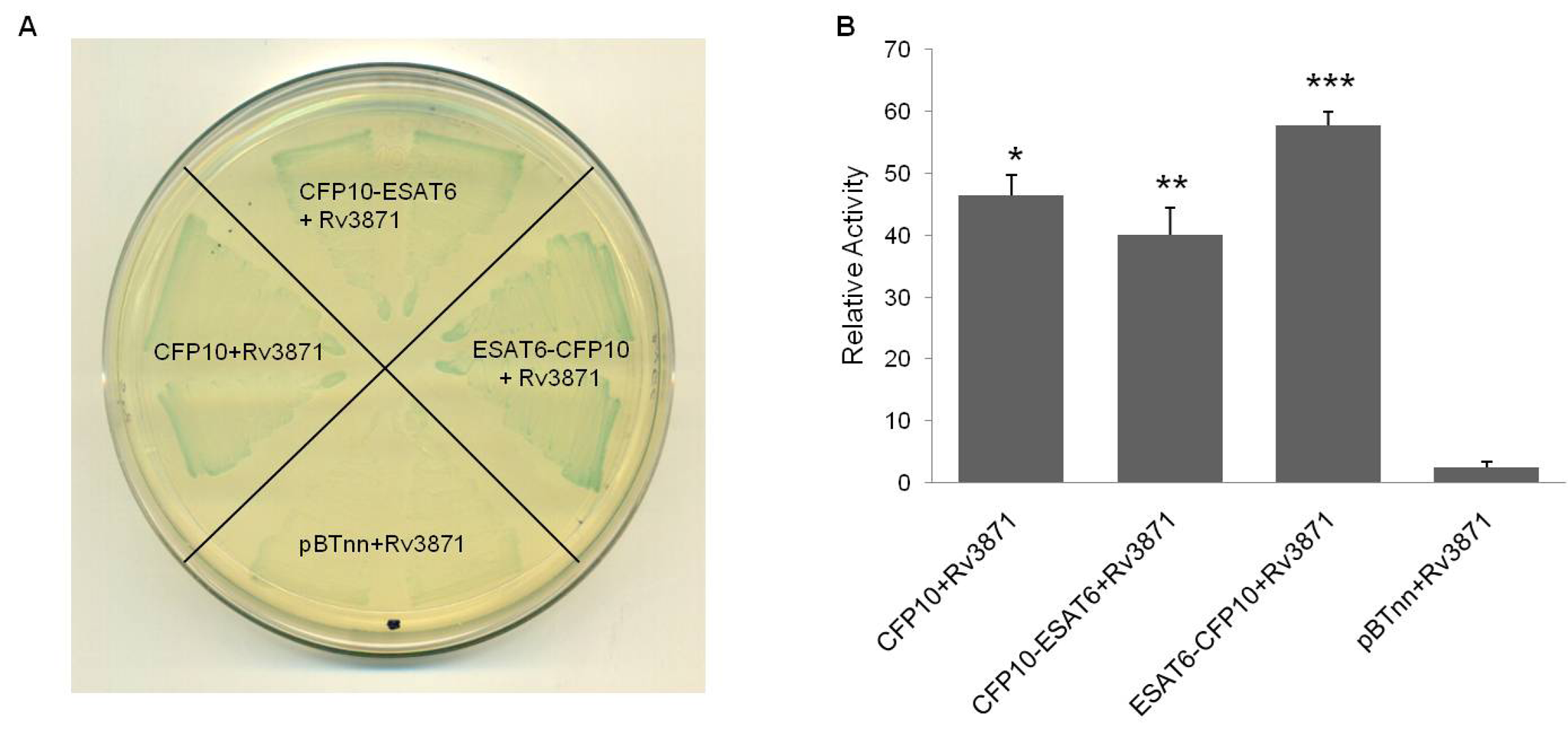

Supplement: Figure S3 — Representation of protein-protein interaction of the CFP10 and ESAT6 fusion constructs with Rv3871 in bacterial two-hybrid system. (A) Bacterial two-hybrid X-Gal plate showing co-transformants CFP10pBTnn + Rv3871pTRGnn; CFP10-ESAT6pBTnn + Rv3871pTRGnn; ESAT6-CFP10pBTnn + Rv3871pTRGnn; and pBTnn + Rv3871pTRGnn (negative control). Two different colonies of each co-transformant were patched (B) Quantitative analysis by liquid β-galactosidase assay. The graph is the average of three separate assays and standard deviation is represented as error bars. (*, P<0.02; **, P<0.05; ***, P<0.01). (TIF) [file pone.0027503.s003.tif]

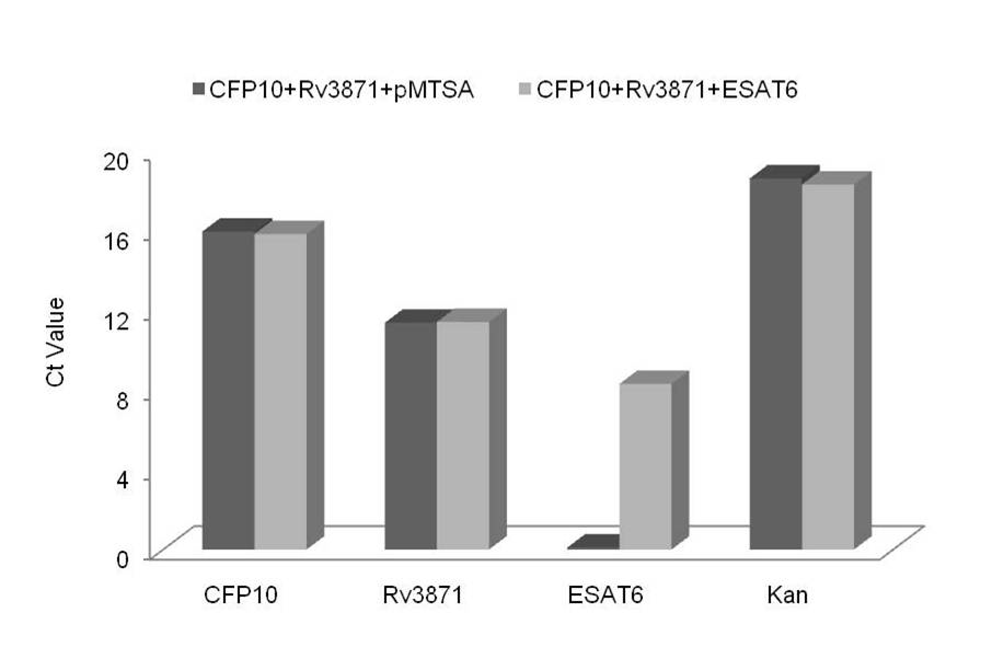

Supplement: Figure S4 — RT-PCR analysis to confirm equivalent expression of CFP10 and Rv3871 in the ESAT6 positive and negative three-hybrid strains. No difference in the transcription level of CFP10 and Rv3871 was observed in the three-hybrid strains CFP10pBTnn+Rv3871pTRGnn+ESAT6pMTSA and CFP10pBTnn +Rv3871pTRGnn+pMTSA indicating that the gradation in the colony blue color of the two strains was solely due to the differential interaction of CFP10 and Rv3871 in the strains influenced by the presence or absence of ESAT6. Kanamycin was used as the internal control. The graph shows an average of three separate assays. (TIF) [file pone.0027503.s004.tif]

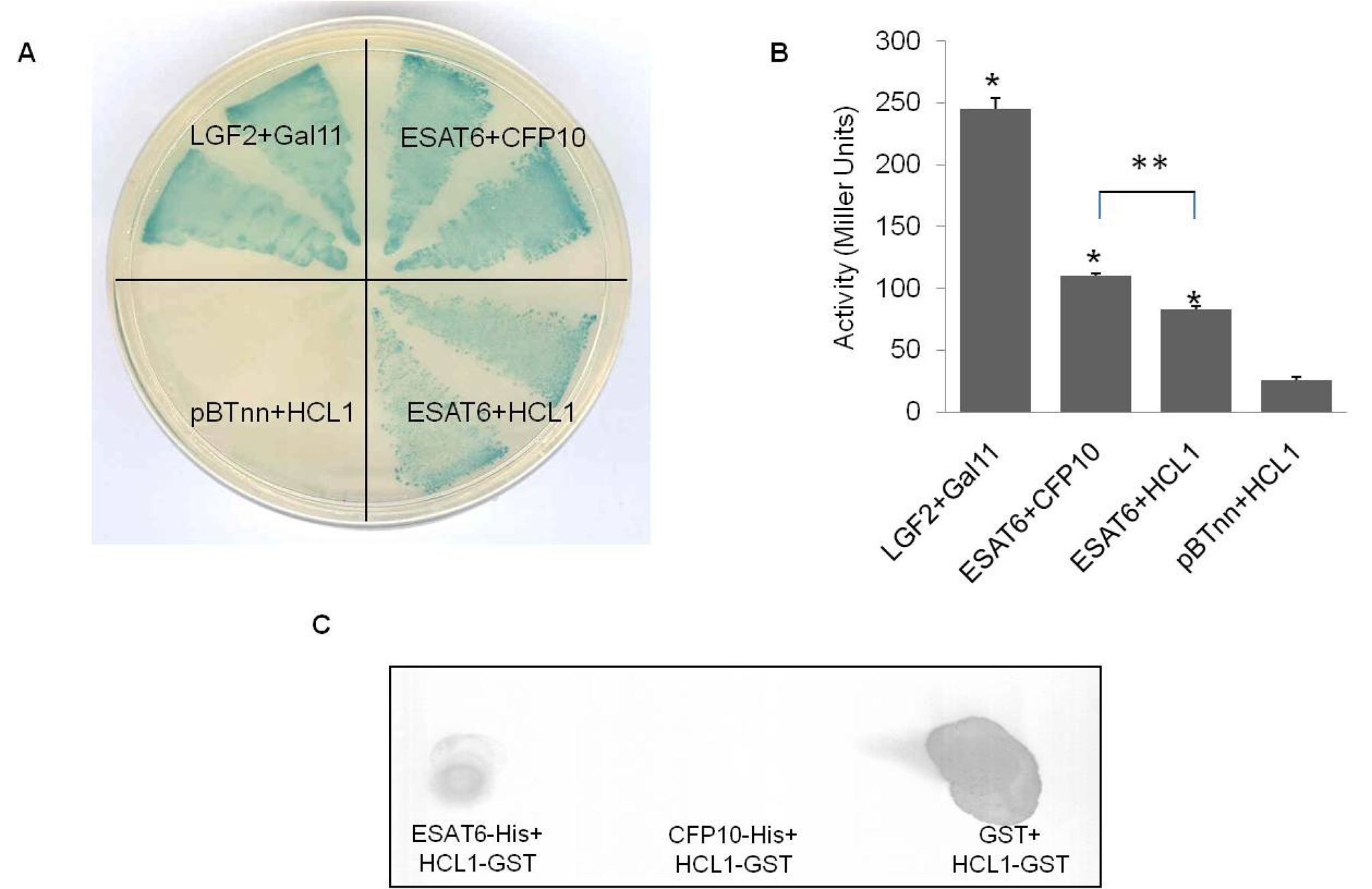

Supplement: Figure S5 — ESAT6 : HCL1 protein-protein interaction. (A) Bacterial two-hybrid X-Gal plate patched with two separate colonies each, of co-transformants LGF2pBTnn + Gal11pTRGnn (positive control); ESAT6pBTnn + CFP10pTRGnn; ESAT6pBTnn + HCL1pTRGnn; and pBTnn + HCL1pTRGnn (negative control) (B) Quantitative representation by liquid β-galactosidase assay. The graph is the average of three independent assays and standard deviation is represented as error bars. (*, P<0.005; **, P<0.02) (C) Far Western Dot Blot Assay: 1 µg each of purified proteins ESAT6-His, CFP10-His (negative control), and GST (positive control) were spotted on nitrocellulose membrane and incubated with 1 µg/mL solution of purified HCL1-GST protein. Blot was developed using anti-GST antibodies. (TIF) [file pone.0027503.s005.tif]

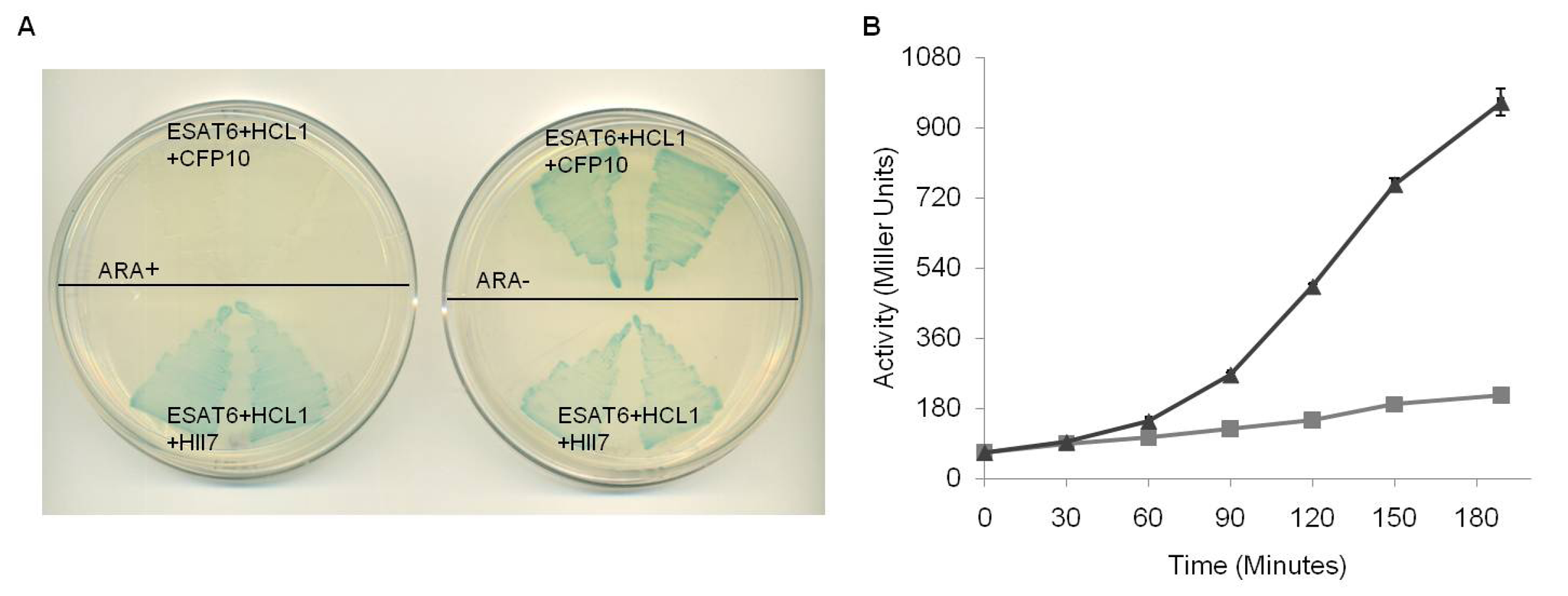

Supplement: Figure S6 — Representation of disruption of ESAT6 : HCL1 protein-protein interaction by CFP10 in bacterial three-hybrid system. (A) X-Gal indicator plate with and without arabinose patched with ESAT6pBTnn + HCL1pTRGnn + HLL7pMTSA; and ESAT6pBTnn + HCL1pTRGnn + CFP10pMTSA. Blue colony turns white when CFP10 is allowed to express in the presence of 1% arabinose while no effect on colony color on expression of the dummy non-interacting peptide HLL7 is seen (B) Time course liquid β-galactosidase assay: β-galactosidase activity of the triple co-transformants: (▴) ESAT6pBTnn + HCL1pTRGnn + HLL7pMTSA; and (▪) ESAT6pBTnn + HCL1pTRGnn + CFP10pMTSA is plotted against time-points of bacterial culture growth with 0 time-point being the point of arabinose induction. Standard deviation of the activities obtained in three separate assays is shown by error bars. (P<0.01 at all time-points beyond 90 minutes). (TIF) [file pone.0027503.s006.tif]

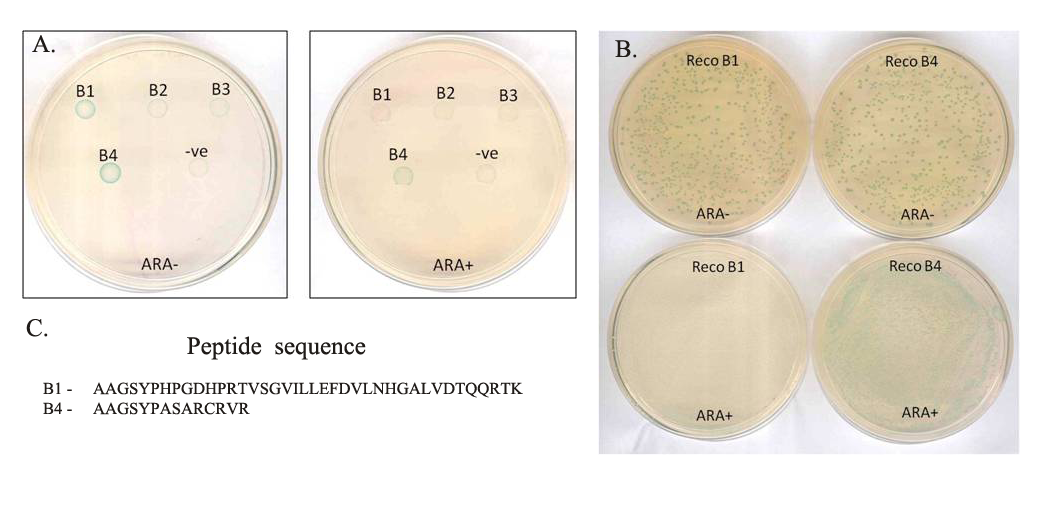

Supplement: Figure S7 — Discovery of a peptide that facilitates the formation of a tri-protein complex. (A) Patching of colonies B1-4 on Arabinose positive and negative plates. B4 remains blue on both plates. (B) Re-cotransformation of mCER1 competent cells with pTRGnn-based library members B1 and B4. RecoB4 remains blue on both Arabinose negative as well as on Arabinose positive plates. (C) Peptide sequences of B1 and B4. (TIF) [file pone.0027503.s007.tif]
